# Bioinformatic Resources for Exploring Human–virus Protein–protein Interactions Based on Binding Modes

**DOI:** 10.1093/gpbjnl/qzae075

**Published:** 2024-10-15

**Authors:** Huimin Chen, Jiaxin Liu, Gege Tang, Gefei Hao, Guangfu Yang

**Affiliations:** State Key Laboratory of Green Pesticide, International Joint Research Center for Intelligent Biosensor Technology and Health, Central China Normal University, Wuhan 430079, China; State Key Laboratory of Green Pesticide, International Joint Research Center for Intelligent Biosensor Technology and Health, Central China Normal University, Wuhan 430079, China; State Key Laboratory of Green Pesticide, International Joint Research Center for Intelligent Biosensor Technology and Health, Central China Normal University, Wuhan 430079, China; State Key Laboratory of Green Pesticide, International Joint Research Center for Intelligent Biosensor Technology and Health, Central China Normal University, Wuhan 430079, China; State Key Laboratory of Green Pesticide, Key Laboratory of Green Pesticide and Agricultural Bioengineering, Ministry of Education, Center for Research and Development of Fine Chemicals, Guizhou University, Guiyang 550025, China; State Key Laboratory of Green Pesticide, International Joint Research Center for Intelligent Biosensor Technology and Health, Central China Normal University, Wuhan 430079, China

**Keywords:** Bioinformatic resource, Viral pandemic, Protein–protein interaction, Artificial intelligence, Protein–protein docking

## Abstract

Historically, there have been many outbreaks of viral diseases that have continued to claim millions of lives. Research on human–virus protein–protein interactions (PPIs) is vital to understanding the principles of human–virus relationships, providing an essential foundation for developing virus control strategies to combat diseases. The rapidly accumulating data on human–virus PPIs offer unprecedented opportunities for bioinformatics research around human–virus PPIs. However, available detailed analyses and summaries to help use these resources systematically and efficiently are lacking. Here, we comprehensively review the bioinformatic resources used in human–virus PPI research, and discuss and compare their functions, performance, and limitations. This review aims to provide researchers with a bioinformatic toolbox that will hopefully better facilitate the exploration of human–virus PPIs based on binding modes.

## Introduction

**Figure 1:**
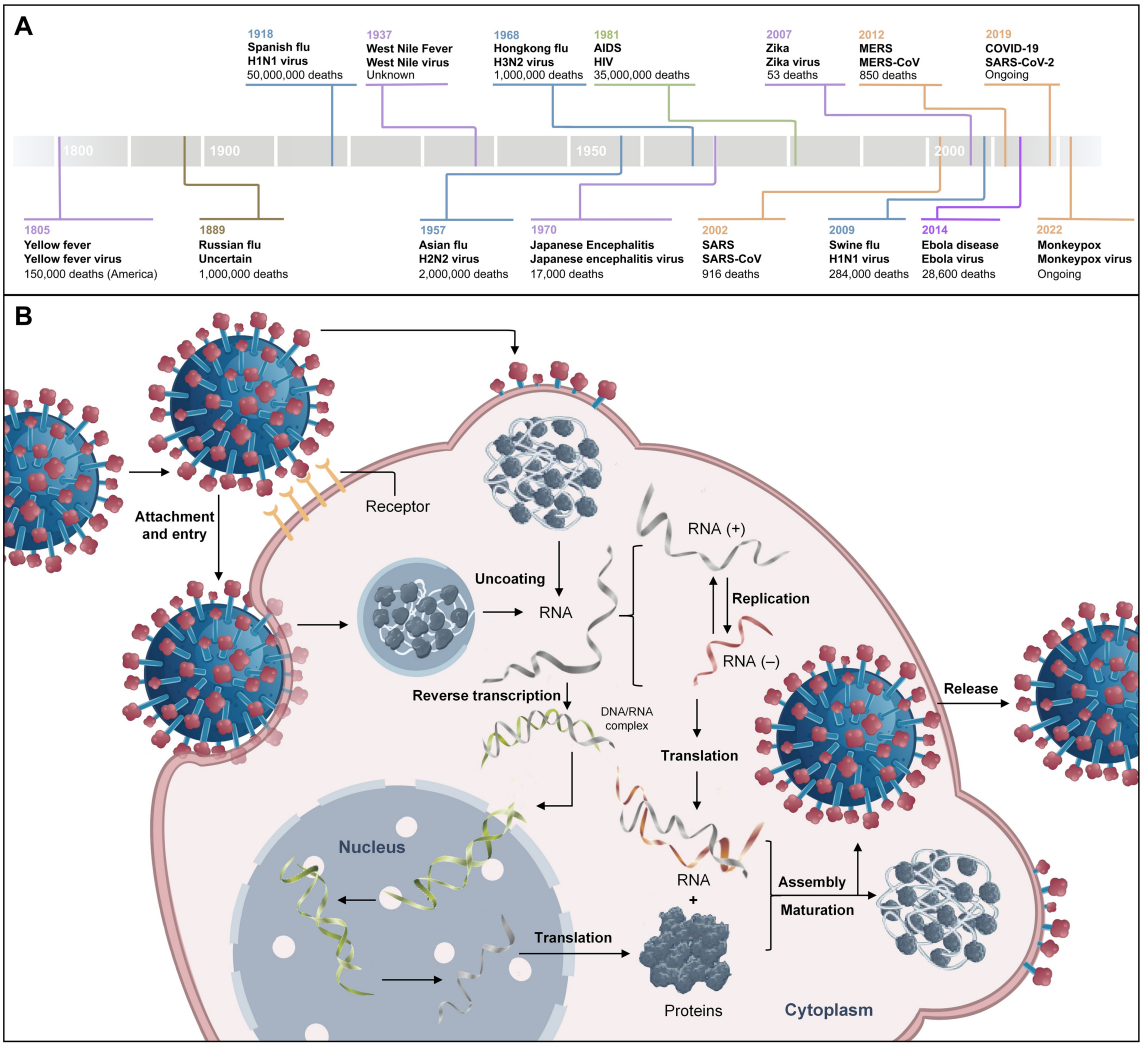
Representative outbreaks of highly pathogenic viruses and the brief life cycle of viruses **A**. The deadliest viral epidemics from 1800 to 2023. Flavivirus epidemics, coronavirus epidemics and influenza A virus epidemics are marked in purple, yellow, and blue, respectively. **B**. Brief life cycle of most enveloped viruses: (1) attachment and entry; (2) uncoating; (3) translation of enzymes; (4) RNA replication; (5) translation of components; and (6) assembly and release. This figure draws on a previous publication [11]. H1N1 virus, influenza A virus subtype H1N1; H2N2 virus, influenza A virus subtype H2N2; H3N2 virus, influenza A virus subtype H3N2; AIDS, acquired immunodeficiency syndrome; HIV, human immunodeficiency virus; SARS, severe acute respiratory syndrome; SARS-CoV, severe acute respiratory syndrome coronavirus; MERS, Middle East respiratory syndrome; MERS-CoV, Middle East respiratory syndrome coronavirus; SARS-CoV-2, severe acute respiratory syndrome coronavirus 2.

In the course of human history, various viral diseases have swept through different parts of the world many times, constantly threatening human health and life. From 1800 to 2024, there were multiple pandemic outbreaks triggered by viruses (**Figure 1**A), including influenza A viruses (IAVs), human immunodeficiency virus (HIV), Dengue virus (DENV), hepatitis C virus (HCV), and severe acute respiratory syndrome coronavirus 2 (SARS-CoV-2) [1–3]. Despite substantial advances in our health infrastructure and knowledge to control infectious diseases, new viral disease-causing threats are constantly emerging [4,5]. Infectious diseases account for 20% of global mortality, with approximately one-third of deaths attributed to viral infections [6,7]. Therefore, it is crucial to explore the human–virus relationships.

The critical entry site for the complex human–virus relationships is deciphering human–virus protein–protein interactions (PPIs) [8,9]. Viral infection of host cells, completion of its replication, and escape from host immunity greatly rely on host factors, and this process is dependent on human–virus PPIs (Figure 1B) [10,11]. Haas et al. [12] used affinity purification-mass spectrometry (AP-MS) to identify 332 human–IAV PPIs, and further global proteomic analysis identified 13 kinases regulated in IAV infection. Zhou et al. [13] revealed 361 novel high-confidence human–SARS-CoV-2 PPIs, based on which 23 drugs with remarkable network proximity to SARS-CoV-2 host proteins were identified. Research of human–virus PPIs contributes to a more profound knowledge of the nature of life activities and the mechanism of viral infections and provides targets for antiviral drug development.

In recent years, the dramatic expansion of data on human–virus PPIs resulting from the emergence of proteomics-based novel methods has created favorable conditions for developing bioinformatic resources for human–virus PPIs. For example, BioGRID [14] is dedicated to the collection of experimentally supported PPIs, which contain 33,520 human–virus PPIs. BioGRID provides datasets of human–virus PPIs for different viral species. During the Coronavirus disease 2019 (COVID-19) outbreak, P-HIPSTer [15] predicted 15 coronaviruses with different pathogenic potentials, reporting 4587 PPIs covering 397 human proteins. VirHostNet [16] provides scientists with a collection of human–SARS-CoV-2 PPIs in near real-time. The emergence of these bioinformatic resources enable accelerated effective tracking of the underlying biological problems. However, a comprehensive summary of the web tools used to explore the human–virus PPIs is unavailable.

In this work, we survey the mainstream web tools available to explore human–virus PPIs. First, human–virus PPI databases are emphasized and elaborated regarding functionality, data volume, and data redundancy. Second, we briefly describe the use of web servers and how they advance research specifically on human–virus PPIs. Finally, we summarize and compare the performance of protein–protein docking tools on human–virus PPI data. Our review may help guide biomedical, chemical, and pharmaceutical researchers to take advantage of appropriate bioinformatic resources for studying human–virus PPIs to promote the development of more advanced bioinformatic tools to improve drug discovery efficiency.

## PPI data for exploring mechanisms of virus pathogenicity

Exploring human–virus PPIs is critical for knowing the precise sequence of events governing the cellular response to infection and mediating the viral replication cycle. However, our knowledge of the mechanisms that mediate and control host–virus interactions remains sparse. Considerable effort has been invested in delineating human–virus PPIs using various methods, including yeast two-hybrid [13,17] and AP-MS [18,19]. These approaches provide a significant amount of data on human–virus PPIs that promote the establishment of databases of human–virus PPIs and yield critical insights into human–virus relationships, identifying immune-critical mediators, and discovering cellular factors that control viral replication [20].

Human–virus PPI databases are categorized according to viral species into specific viral species and pan-viral species databases. The HIV-1 Human Interaction Database (HHPID) [21] is one of the most representative specific viral species databases, storing all known information on human–HIV-1 interactions, including human–HIV-1 PPIs, proteins from HIV/AIDS-related disease organisms, and human genes that affect viral replication and infectivity. HCVpro [22] is a comprehensive HCV-specific knowledge database providing complete information on PPIs, molecular data, and functional genomics. In addition, HCVpro provides information on hepatocellular carcinoma-associated genes. DenHunt [23] is an integrated database designed for human–DENV PPIs. However, databases designed for specific viral strains do not fulfill the needs of a broader range of researchers. Several pan-species databases have been developed for this purpose. DIP [24] is the earliest database to incorporate experimentally validated PPIs, including human–virus PPIs. DIP also provides quality evaluation approaches to assess the reliability of PPIs. BioGRID [25] and IntAct [26] are comprehensive public databases of PPIs. BioGRID specializes in model organism PPIs and provides post-translational modifications (PTMs) and bioactive small molecule interactions. In addition, IntAct provides molecular interaction (MI) scores for interaction relationships and interaction analysis for nucleic acids such as miRNA and lncRNA. VirHostNet, VirusMentha [27], and Viruses.STRING [28] are comprehensive PPI resources focusing on host–virus PPI data. Owing to the wide range of data sources for PPIs, Viruses.STRING provides a confidence score that measures the true probability of PPIs based on several different sources of PPIs. In contrast, HPIDB [29] and PHISTO [30] focus on containing more host–pathogen PPIs, but only provide basic information about the PPIs, such as UniProt IDs and detection methods, in their entries. HVIDB [31] and HVPPI [32] focus on human–virus PPI databases, providing fully annotated information on human–virus PPIs.

To further understand the human–virus PPI databases, a comparison was performed among the functions of the previously mentioned databases (**Table 1**). All databases except HHPID and HCVpro have PPI network visualization to render sophisticated inter-relationships seem intuitive. Notably, HVPPI provides information and visualization of PPI and drug–target interaction data. HPIDB and VirHostNet built BLAST-DB based on their own PPI data, providing a homologous fast search of human–virus PPIs. Different Gene Ontology (GO) and Kyoto Encyclopedia of Genes and Genomes (KEGG) enrichment analyses were provided by PHISTO, HVIDB, and Viruses.STRING. HVIDB and HVPPI provide human–virus PPIs and viral protein function prediction tools, respectively.

**Table 1:** Databases for human–virus PPIs (data up to Feb 2024)

Database/URL	No. of VFs	Statistics				Functional annotation	Online analysis tool (additional services)	Main data source	Data construction method
		No. of host–virus PPIs	No. of human–virus PPIs	No. of human–SARS-CoV-2 PPIs	No. of plant–virus PPIs				
**Specific viral species databases**									
HHPID https://www.ncbi.nlm.nih.gov/genome/viruses/retroviruses/hiv-1/interactions/	1	6824	6824	–	–	–	–	–	Publications
HCVPro https://www.cbrc.kaust.edu.sa/hcvpro/	1	549	549	–	–	GO/KEGG annotations, Reactome pathway, Pfam, Interpro, subcellular localization, domain	–	VirHostNet, VirusMint, HCVdb, euHCVdb, BIND	Database integration, publications
DenHunt http://proline.biochem.iisc.ernet.in/DenHunt/	1	682	682	–	–	KEGG annotations, host factors	PPI network visualization	–	Publications
**Pan-viral species databases**									
DIP https://dip.doe-mbi.ucla.edu/dip/Main.cgi	19	574	574	–	–	GO annotations, Pfam, Interpro, SMART, Prosite, PRINTS	BLAST, PPI network visualization, motif, EPR index, PVM score, DPV score	–	Publications
BioGRID https://thebiogrid.org/	6	33,520	26,917	18,769	3	GO annotations, PTM, host factors	PPI network visualization	–	Publications
IntAct https://www.ebi.ac.uk/intact/home	–	31,718	27,134	491	–	GO annotations	PPI network visualization, MI score	–	Publications
VirHostNet https://virhostnet.prabi.fr/	31	48,664	40,416	6731	–	GO annotations, host factors	BLAST, PPI network visualization	IntAct, Mint, DIP, InnateDB, BIND, UniProt, HPIDB, Viralzone	Database integration, publications
VirusMentha https://virusmentha.uniroma2.it/	27	15,967	10,692	–	∼ 17	GO/KEGG annotations	PPI network visualization	MINT, IntAct, DIP, MatrixDB, BioGRID	Database integration, publications
Viruses.STRING http://viruses.string-db.org/	–	–	–	–	–	GO annotations	PPI network visualization, confidence score	IntAct, STRING, HPIDB, BioGRID, VirusMentha	Database integration, text mining
HPIDB https://hpidb.igbb.msstate.edu/hpi30_index.html	34	39,506	38,669	–	54	–	BLAST, PPI network visualization	IntAct, MINT, UniProtKB, Molecular Connections, MBInfo, I2D, MPIDB, InnateDB, BioGRID, BIND, DIP, MatrixDB, VirHostNet	Database integration, publications
PHISTO https://www.phisto.org/index.xhtml	34	39,558	39,621	–	–	–	PPI network visualization, GO/KEGG enrichment, graph analysis	APID, IntAct, DIP, MINT, iRefIndex, Viruses.STRING, MPIDB, BIND, Reactome	Database integration
HVIDB http://zzdlab.com/hvidb/	35	48,643	48,643	303	–	GO/KEGG annotations, homologous, subcellular localization, domain, SNP, host dependency/restriction factors	PPI network visualization, GO/KEGG enrichment, prediction of human–virus PPIs, differentially expressed genes post viral infections	HPIDB, PHISTO, VirHostNet, VirusMentha, PDB	Database integration, publications
HVPPI http://bio-bigdata.hrbmu.edu.cn/HVPPI/	8	27,293	27,293	755	–	GO annotations	PPI network visualization, drug–target interaction network visualization	HPIDB, HVIDB, VirusMentha, VirHostNet, DenHunt, HCVPro, GPS-Prot, DenvInt, hu.MAP2.0	Database integration, publications

*Note*: PPI, protein–protein interaction; VF, virus family; SARS-CoV-2, severe acute respiratory syndrome coronavirus 2; GO, Gene Ontology; KEGG, Kyoto Encyclopedia of Genes and Genomes; SNP, single-nucleotide polymorphism; PTM, post-translational modification; EPR, expression profile reliability; PVM, paralogous verification; DPV, domain pair verification, MI, molecular interaction.

**Figure 2:**
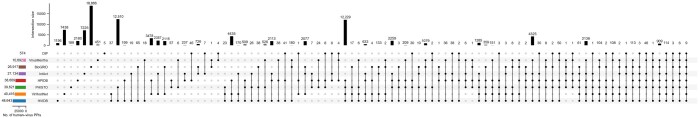
Comparison of human–virus PPI data in databases The bar graph above indicates the number of redundancies of human–virus PPIs in different databases; the horizontal bar graph on the lower left side indicates the number of human–virus PPIs contained in each database; the bitmap on the lower right side indicates the way different databases overlap and combine. The number of redundancies is defined as the number of repeated appearances of the same data in different databases. PPI, protein–protein interaction.

We further compared the data volume and the data sources of the databases. VirHostNet has 48,664 host–virus PPIs, which is the largest dataset of experimentally validated PPIs about viruses. Meanwhile, HVIDB is the largest dataset of human–virus PPIs with 48,643 experimentally validated human–virus PPIs. We analyzed the data comparatively and found that data on host–virus PPIs mainly focus on human–virus PPIs, and that only BioGRID, VirusMentha, and HPIDB contain data on a minimal number of plant–virus PPIs (Table 1). Subsequently, by comparing the data on human–virus PPIs in different databases, we found that BioGRID, VirHostNet, and IntAct have more human–virus PPI data that are non-redundant, which are 18,866, 7438, and 7225, respectively (**Figure 2**). In contrast, HVIDB has the largest dataset of human–virus PPIs, but only 1156 PPIs are non-redundant. Further combining the data sources revealed that most human–virus PPI data in VirusMentha, Viruses.STRING, PHISTO, HVPPI, and HVIDB are derived from human–virus PPIs in other databases, leading to significant redundancy and interdependence. HVIDB depends on HPIDB, PHISTO, and VirusMentha. PHISTO depends on VirusMentha and Viruses.STRING. HPIDB depends on VirusMentha. HVPPI depends on HPIDB, HVIDB, and VirusMentha. BioGRID, VirHostNet, and IntAct focus more on integrating PPIs from the literature, so other databases largely depend on these databases for their data.

Based on the abovementioned analysis, the currently available human–virus PPI databases remain to be enhanced. First, databases use various formats, making it difficult for users to download, analyze, and visualize data from multiple sources in a standard format [20]. Second, the data on human–virus PPIs in the databases still need to be completed, and PPIs for some virus families are even blank. Third, most of these databases fail to offer any drug-related information that would help advance antiviral therapy further. Finally, designing and managing a benchmark dataset of human–virus PPIs is necessary for better selection of different prediction tools for various situations. The human–virus PPI databases need to be adapted to the new era of big data as soon as possible.

## Computational resources accelerate the discovery of new PPIs

Human–virus PPI data help drive the discovery of novel PPIs and are essential for comprehending human–virus relationships [33]. However, the scalability constraints of high-throughput approaches hinder the large-scale identification of human–virus PPIs [34]. Indeed, of the approximately 1000 unique viruses that infect humans, only a handful of human–virus PPIs are well studied. Despite their indisputable public health importance, very little is known about most viruses beyond their genome sequences [35,36]. Hence, computational prediction approaches are becoming progressively crucial for complementing experimental work. Existing prediction methods include inference based on domain–domain interactions (DDIs), interolog mapping, and others. More information on these computational approaches can be found in the reviews [37,38]. Here, we focus more on machine learning (ML)-based techniques for predicting human–virus PPIs. Notably, most of these sequence-based ML prediction tools for human–virus PPIs have model features learning from the interaction networks of the training and test sets, rather than from protein sequences [39].

Several predictive tools have been developed using traditional ML methods (**Table 2**). hivPPI [40] is the first effective prediction ML model for human–HIV-1 PPIs that integrates multiple biological information source features (*e.g.*, GO annotations). In contrast to the underfitting of various features, Cui et al. [41] proposed a new feature representation method that employs frequency vectors at a fixed length to represent variable-length protein sequences. However, these prediction methods are mainly designed for specific viruses, which significantly limits their adaptability. Barman et al. [42] introduced the first support vector machine (SVM)-based prediction model for human–virus PPIs to conquer the limitations of the adaptability of prediction methods. Subsequently, considering the noise of random negative sampling, DeNovo [43] proposes noise reduction by a negative sampling approach based on dissimilarity, which utilizes shared host proteins and learns from different virus PPIs to predict novel viruses. VirusHostPPI [44] uses a multi-feature fusion approach to merge six features representing protein sequences, including the frequency difference of amino acid triplets (FDAT), the relative frequency of amino acid triplets (RFAT), amino acid composition (AC), and the composition, transition, and distribution of amino acid groups. Although these feature codes consider the specific physicochemical properties or interaction effects of the residues to a certain extent, they cannot adequately consider the semantic information in the whole sequence. Therefore, using doc2vec for the first time to predict human–virus PPIs, HVPPI [45] dramatically enhances the accuracy rate. In contrast, HVIDB integrates three different models, including internal mapping, DDIs, and random forest (RF), to construct prediction models via logistic regression. PrePPI [46] uses a Bayesian framework to predict PPIs, and it shows that 3D structures are better than non-structural methods for predicting PPIs. The P-HIPSTer further exploits the structure information of human–virus complexes based on the PrePPI algorithm to predict human–virus PPIs by considering both peptide–domain interactions and DDIs. The accuracy of P-HIPSTer is nearly 80%, but it is not available for online prediction.

**Table 2:** Prediction servers for human–virus PPIs

Server/URL	Feature engineering	Model architecture	Negative sampling	Positive sample source	No. of positive/negative samples	Performance evaluation strategy
hivPPI www.cs.cmu.edu/∼oznur/hiv/hivPPI.html	Sequence (sequence similarity), network, biological function (GO, domain-motif, PTM), expression (gene expression, tissue expression)	RF	Random	NIAID	1063/106,300	3-fold cross-validation
DeNovo https://bioinformatics.cs.vt.edu/∼alzahraa/denovo	Amino acid triplet	SVM	Dissimilarity-based	VirusMentha	5447/5161	5-fold cross-validation
VirusHostPPI http://165.246.44.47/VirusHostPPI/	RFAT, FDAT, AC, the composition, transition, and distribution of amino acid groups	SVM	CD-HIT-2D < 80%	APID, IntAct, VirusMentha	12,158 (11,491 human–virus PPIs)/12,158, 24,316, 36,474	10-fold cross-validation
HVPPI http://zzdlab.com/hvppi/	doc2vec	RF	Random	HPIDB	22,653/226,530	5-fold cross-validation
HVIDB http://zzdlab.com/hvidb/predict.php	doc2vec	Interolog, DDI, RF	Dissimilarity-based	HVIDB	31,383/313,830	Independent test
PrePPI http://bhapp.c2b2.columbia.edu/PrePPI	–	Bayesian network	–	MIPS, DIP, IntAct, MINT, HPRD, BioGRID	199,863/–	5-fold cross-validation
P-HIPSTer http://phipster.org/	–	Bayesian network	–	Virus-hostDB, UniProt	12,237/–	5-fold cross-validation
PIPR https://github.com/muhaochen/seq_ppi	RCNN	GRU	–	STRING, SKEMPI	26,945/–	5-fold cross-validation
deepHPI http://bioinfo.usu.edu/deepHPI/	PAAC, CT, NMBroto	CNN	Neglog	HPIDB	42,491/424,910	5-fold cross-validation
LSTM-PHV http://kurata35.bio.kyutech.ac.jp/LSTM-PHV/	doc2vec	LSTM, MLP	Dissimilarity-based	HPIDB	22,383/223,830	Independent test
DeepViral https://github.com/bio-ontology-research-group/DeepViral	One-hot, node2vec	CNN, MLP	Random	HPIDB, PathoPhenoDB	24,678/246,780	Leave-one-family-out cross validation
TransformerGO https://github.com/Ieremie/TransformerGO.	node2vec	Transformer	Random	Jain’s datasets	420,534/4,205,340	5-fold cross-validation
TransPPI https://github.com/XiaodiYangCAU/TransPPI/	PSSM	CNN, MLP, transfer learning	Dissimilarity-based	HPIDB, VirHostNet, VirusMentha, PHISTO	31,381/313,810	5-fold cross-validation
DeepVHPPI https://github.com/QData/DeepVHPPI	One-hot	CNN, MLP, transfer learning	Dissimilarity-based	HPIDB	22,653/226,530	Independent test
MTT https://git.l3s.uni-hannover.de/dong/multitask-transfer	mLSTM	MLP, transfer learning	Multiple settings	Multiple settings	Multiple settings	5-fold cross-validation
TAGPPI https://github.com/xzenglab/TAGPPI	Amino acid embedding, graph learning	CNN	Random	Uniref50	–	5-fold cross-validation
Struct2Graph https://github.com/baranwa2/Struct2Graph	GCN	Mutual attention network	Dissimilarity-based	IntAct, STRING	4698/112,353	5-fold cross-validation

*Note*: RFAT, relative frequency of amino acid triplets; FDAT, frequency difference of amino acid triplets; AC, amino acid composition; RCNN, recurrent convolutional neural network; PAAC, pseudo amino acid composition; PSSM, polysaccharide storage myopathy; mLSTM, multiplicative long short-term memory; GCN, graph convolutional network; RF, random forest; SVM, support vector machine; DDI, domain–domain interaction; CNN, convolutional neural network; MLP, multi-layer perceptron; CT, conjoint triad; NMBroto, normalized Moreau-Broto autocorrelation; GRU, gated recurrent unit; LSTM, long short-term memory.

Deep learning (DL), an essential branch of ML, can effectively complement traditional ML methods by permitting flexibility in allowing known labels and feature inputs compared to traditional ML methods (Table 2). PIPR [47] is an end-to-end prediction framework based on recurrent regression convolutional neural network (RCNN), which offers an automatic multi-granularity feature selection mechanism to learn sequential and locally significant features of primary protein sequences. deepHPI [48] provides predictions for four host–pathogen models based on a convolutional neural network (CNN) model architecture: animal–pathogen, human–virus, plant–pathogen, and human–bacteria. Although CNN can better capture local features of protein sequences, there is a problem of gradient explosion and disappearance. LSTM-PHV [49] utilizes long short-term memory to overcome this problem, thus effectively learning of long-sequence proteins. DeepViral [50] and TransformerGO [51] use the node2vec for learning continuous representations of PPI network nodes, which are then used as input for training neural networks. However, its dependence on node information features (*e.g.*, GO information) may constrain its applicability. To address the relative scarcity of data on virus species and improve the prediction model’s generalization ability, MTT [52], DeepVHPPI [53], and TransPPI [54] have further introduced transfer learning to the prediction of human–virus PPIs. TAGPPI [55] enhances the performance of sequence-based prediction methods by sequence features with structural information predicted by AlphaFold into PPI prediction models. Struct2Graph [56] is a graph convolutional network (GCN)-based interaction classifier that predicts PPIs based on 3D structural information. Nevertheless, the lack of 3D protein structures and associated information can also constrain the applicability of the approach.

**Figure 3:**
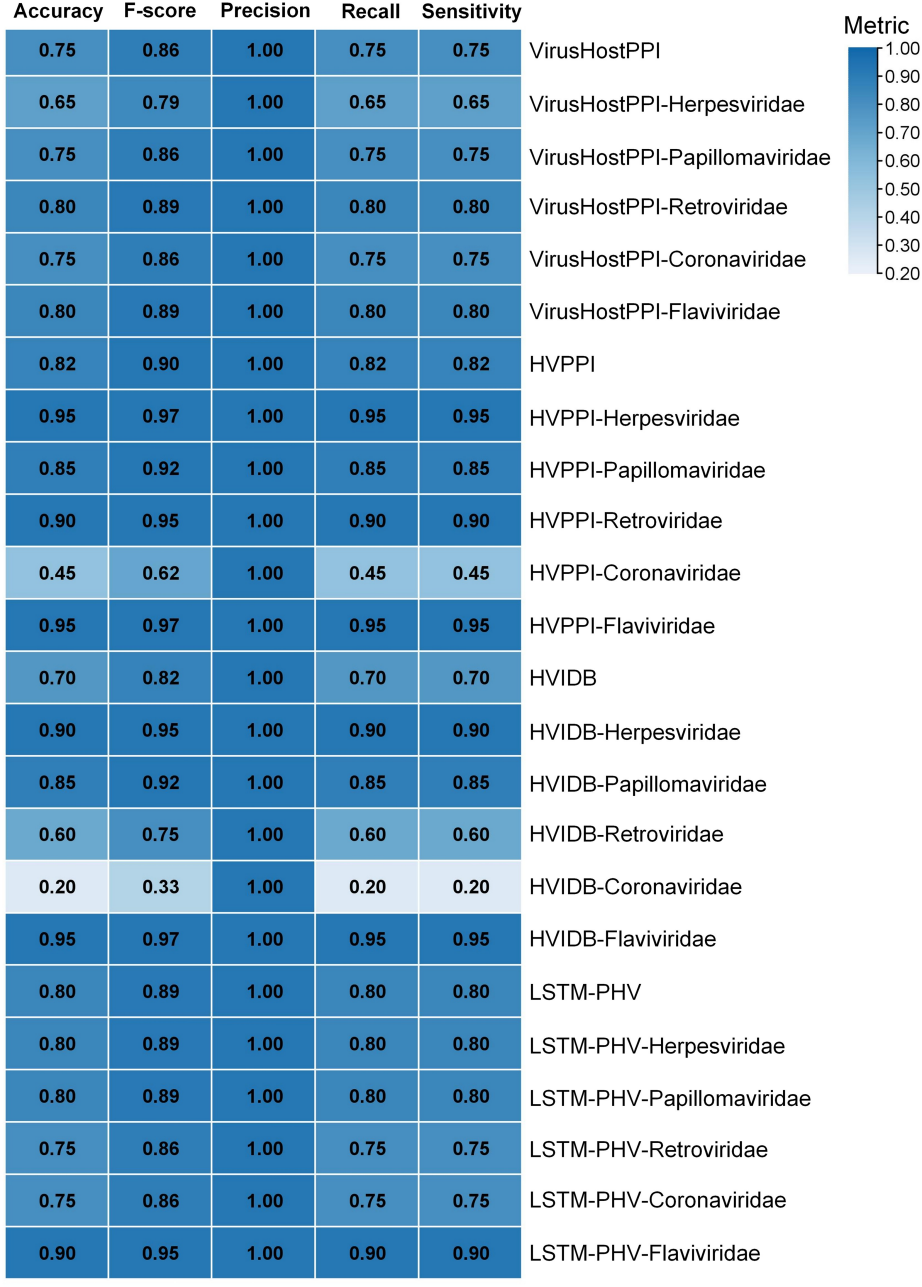
Performance comparison of human–virus PPI prediction tools

To further understand the differences in the performance of these prediction tools, we compared their accuracy. A “gold standard” human–virus PPI dataset is currently lacking, and fair performance comparisons between different prediction methods remain a provocative task [57]. Hence, we collected a positive independent test set (100 non-redundant human–virus PPIs containing four virus families) from VirHostNet to compare the performance of four tools, VirusHostPPI, HVPPI, HVIDB, and LSTM-PHV (**Figure 3**, Figure S1; Table S1). It is worth noting that we use accuracy to refer to the true positive rate since the benchmark set contains only positive PPIs. The four tools were selected for evaluation with the following selection criteria: (1) sequence-based prediction; (2) different model architectures; and (3) convenience of tool testing. The accuracy of HVPPI and LSTM-PHV was over 80%, with HVPPI achieving the highest accuracy at 82%. In contrast, HVIDB showed poor performance. Further analysis of the specificity of different virus families showed that the prediction performance of LSTM-PHV and VirusHostPPI was relatively balanced for different virus families. LSTM-PHV achieved more than 75% prediction accuracy for different virus families, with the best prediction performance for Flaviviridae, with 90% accuracy. The overall prediction performance of VirusHostPPI was lower than that of LSTM-PHV, with better prediction performance in Papillomaviridae and Flaviviridae, with 80% accuracy. In contrast, the models for HVPPI and HVIDB were not well adapted and had an uneven prediction performance for different virus families. Predictions for Herpesviridae and Flaviviridae were better, with accuracy above 90%. The prediction accuracy for Coronaviridae was less than 50%. In addition, the accuracy of Coronaviridae prediction was concentrated in the range of 20% to 75%, with a relatively poor overall performance, which may be related to the lack of relevant samples in the training set. It is worth noting that the test samples are limited and thus do not represent broad conclusions. The performance on different virus families needs to be further expanded.

There is still significant potential for improvement, despite the emergence of various human–virus PPI prediction tools. First, constructing a prediction model for human–virus PPIs should introduce more features of the protein sequences. Most of the model features of current sequence-based prediction models are learning from PPI networks or using simple sequence features (*e.g.*, *k*-mers), which do not accurately describe the physical interactions between proteins. Second, the training data of human–virus PPI models are mainly concentrated in specific richly studied virus families (*e.g.*, HIV). Data for most virus families are lacking, and although transfer learning effectively solves the sample size problem, the differences between the data can compromise the model’s specificity. Third, it is difficult to create standard datasets of non-PPIs owing to the difficulty of obtaining experimentally validated negative samples. At present, negative samples are mainly constructed by random sampling or dissimilarity-based negative sampling. However, these methods all misallocate positive samples to negative samples to a certain extent, which can mislead the model learning process and reduce the prediction sensitivity [58]. In addition, several methods were developed to learn features of PPIs from positive samples only, thus avoiding the negative sample construction. Yet, prediction methods that lack learning from negative samples inevitably generate a high risk of false positives. Fourth, a highly unbalanced ratio of data samples (positive samples *vs.* negative samples = 1:10) will cause the model to be biased toward negative sample features when learning. Therefore, adjusting the balance of positive and negative samples remains challenging. Finally, it is crucial to carefully construct benchmark datasets to ensure impartiality when comparing different prediction tools.

## Docking tools for elucidating binding modes of PPIs

Elucidating human–virus PPI binding modes is vital for understanding the molecular mechanisms of the protein–protein recognition [59,60]. Currently, there are many experimental methods for the determination of PPIs, such as AP-MS, and there are also some methods for the prediction of PPIs, such as PrePPI, but it is difficult to rely on the experimental techniques of biophysics or biochemistry alone to reveal the human–virus PPI information at the atomic level. Moreover, the number of crystal structures of human–virus protein complexes in the Protein Data Bank (PDB) remains extremely limited owing to high experimental costs and technical difficulties [61,62]. Thus, it is essential in deriving structural information about human–virus protein dimers and larger complexes through protein–protein docking methods [63,64]. As computational biology continues to develop, various *in silico* tools have been developed to help elucidate the binding modes of human–virus PPIs. Here, we first summarize the available protein–protein docking tools, which according to their algorithms can be classified into two major categories: template-free docking and template-based docking (**Table 3**). Further, to guide the readers in selecting an appropriate docking tool, we evaluated the performance of nine docking tools on human–virus protein complexes.

**Table 3:** Prediction tools for binding modes between human and virus proteins (data up to Feb 2024)

Server/URL	Type	Sampling algorithm	Scoring function	Accuracy provided by relevant references	No. of citations
DOT https://www.sdsc.edu/CCMS/DOT/	Rigid	FFT	Electrostatics, desolvation	Top 30: 50%	482
GRAMM-X https://gramm.compbio.ku.edu/	Rigid	FFT	Electrostatics, desolvation	Top 10: 16% (CAPRI round 18)	984
pyDockWEB https://life.bsc.es/pid/pydockweb	Rigid	FFT	Electrostatics, desolvation	Top 20: 37%; Top 100: 56%	624
ClusPro https://cluspro.org/login.php	Rigid	FFT	Energy	Top 10: 64.28%	4324
ZDOCK http://zdock.umassmed.edu/	Rigid-flexible	FFT	Shape complementarity, electrostatics, knowledge-based pair	Top 1: 12%; Top 50: 51%	3681
MEGADOCK http://www.bi.cs.titech.ac.jp/megadock/index.html	Rigid	FFT	High-throughput	Top 1: ∼ 3%; Top 10: ∼ 10%; Top 100: ∼ 20%; Top 1000: ∼ 28%	215
FRODOCK http://frodock.chaconlab.org	Rigid	FFT, SH	Electrostatics, desolvation, knowledge-based pair	Top 1: 10%; Top 10: 29%; Top 100: 61%; Top 1000: 82%	384
GalaxyTongDock https://galaxy.seoklab.org/cgi-bin/submit.cgi?type=TONGDOCK_INTRO	Rigid	FFT	Energy	Top 1: 17.1%; Top 10: 32.9%; Top 50: 48.7%	37
HDOCK http://hdock.phys.hust.edu.cn/	Rigid-flexible	FFT	Shape complementarity, electrostatics, desolvation	Top 1: 11.1%; Top 10: 29.6%; Top 100: 59.3%; Top 1000: 72.2%	1499
CoDockPP http://codockpp.schanglab.org.cn/	Rigid	FFT	Precise knowledge-based	Top 1: 13.9%; Top 10: 32.2%; Top 100: 57.8%; Top 1000: 80.0%	37
PatchDock https://bioinfo3d.cs.tau.ac.il/PatchDock/bin/htmlInvokePatchDock.pl	Rigid	GH	Shape complementarity	–	3206
LzerD https://lzerd.kiharalab.org/upload/	Rigid	GH	Shape complementarity	Top 1: 40.0%; Top 5: 45.0%; Top 10: 48.7% (CAPRI round 46)	118
SwarmDock https://bmm.crick.ac.uk/∼svc-bmm-swarmdock/	Rigid	PSO	Electrostatics, desolvation	Top 1: 10.8%; Top 5: 29%; Top 10: 36.4%; Top 50: 57.4%; Top 100: 65.3%	300
LightDock https://server.lightdock.org/	Rigid-flexible	GSO	Multi-scale	Top 10: 10%; Top 100: 20%	110
HawkDock http://cadd.zju.edu.cn/hawkdock/	Rigid	ATTRACT: randomized search algorithm	Van der Waals, electrostatics, desolvation	Top 10: 25%; Top 50: 42.31%; Top 100: 50.00%; Top 200: 69.23%; Top 400: 80.77%; Top 1000: 88.46%	363
MDockPP https://zougrouptoolkit.missouri.edu/MDockPP/	Rigid-flexible	Reduced model FFT	Shape complementarity	Top 1: 28.5%; Top 5: 50.0% (CAPRI round 50)	74
RosettaDock http://rosettadock.graylab.jhu.edu/	Rigid-flexible	MC	Electrostatics, desolvation	Top 10: 80% (CAPRI round 5)	629
HADDOCK https://haddock.science.uu.nl/services/HADDOCK2.2/	Rigid-flexible	AIRs	Electrostatics, desolvation	Top 10: 28.5%	6900
PRISM http://cosbi.ku.edu.tr/prism	Rigid	MultiProt: structural comparison engine	Energy	–	203

*Note*: FFT, Fast Fourier Transform; SH, spherical harmonic; GH, genetic algorithm; PSO, Particle Swarm Optimization; GSO, Glowworm Swarm Optimization; MC, Monte Carlo; AIR, ambiguous interaction restraint; CAPRI, Critical Assessment of PRedicted Interactions.

### Protein–protein docking tools

Template-free docking is a docking method that does not require protein structure as a template. It can be categorized into two categories based on the availability of binding site information: global docking and local docking [65]. Global docking exhaustively searches the receptor protein surface to capture the binding modes, most of which are correlated using Fast Fourier Transform (FFT) correlation search algorithms. Despite the similarity of the initial FFT-based global search algorithms, each tool has different filtering steps and scoring functions. DOT [66], FTDOCK [67], and GRAMM-X [68] were the earliest to introduce FFT-based rigid protein–protein docking tools. FTDOCK and DOT use shape complementarity and electrostatic complementarity to evaluate binding modes quickly. In contrast, GRAMM-X focuses more on low-resolution docking. pyDockWEB [69] uses the FTDOCK results as initial sampling and evaluates them with desolvation, electrostatics, and limited Van der Waals contributions. ClusPro [70] successfully applies paired knowledge-based energy to the FFT-based docking method. ZDOCK [71], MEGADOCK [72], and FRODOCK [73] are grid-based protein docking algorithms that use FFT to generate docking conformations in a grid-based 3D space. However, the scoring function of MEGADOCK is much simpler and is thus 7.5 times faster than ZDOCK. FRODOCK further incorporates a spherical harmonic function to speed up the conformational search. GalaxyTongDock [74] is a ZDOCK-based docking approach but with its energy parameters re-optimized. HDOCK [75] uses an improved shape complementary scoring function. In sampling, the scoring of ligand grids considers the contribution of its nearest neighboring receptor grid at the same time as the contribution of other receptor grids. HDOCK also supports amino acid sequences as input. CoDockPP [76] proposes a scoring function based on distance-dependent knowledge based on the observed distribution functions of atomic pairs. The function has native and near-natural structures to enhance its robustness to conformational changes. There are also some docking tools based on other types of search strategies, including PatchDock [77], LzerD [78,79], SwarmDock [80], LightDock [81], HawkDock [82], and MDockPP [83]. PatchDock and LzerD use the geometric hashing function to search for initial conformation, with the resulting conformations sorted by the geometric shape complementary scores. LightDock and SwarmDock use the Particle Swarm Optimization and Glowworm Swarm Optimization algorithms for conformation search, respectively, which are population Swarm Intelligence (SI) algorithms. SI can execute a more efficient search in complex spaces, quite independently of the scoring function to optimize. To balance between computational efficiency and accuracy, HawkDock introduces molecular mechanics/generalized Born Surface Area (MM/GBSA) to calculate desolvation potentials. HawkDock also provides an analysis of key residues at the interface of PPIs. However, local docking searches for protein–binding modes based on user-defined binding sites. RosettaDock [84] and HADDOCK [85] are both representative local docking methods that typically perform local protein docking where binding site information is known. They can handle structural flexibility, but RosettaDock can only be used at the side chain level, while HADDOCK allows for full structure. In contrast, template-based docking needs a template, using the structure of similar complexes to predict the binding structure of protein complexes, such as PRISM [86]. Although there have been some successes, template-based docking methods are restricted by known templates.

### Evaluation of docking tools on a human–virus complex dataset

#### Independent test set and docking protocol

To systematically evaluate the performance of docking tools on human–virus complexes, an independent dataset consisting of 50 human–virus protein complexes was constructed, ranging from 5–300 residues in length for viral proteins. This independent test set was extracted from the PDB and HVIDB. The extraction criteria were as follows: (1) virus protein length ranges from 5 to 300 residues; (2) the resolution ≤ 2.0 Å; and (3) the protein does not contain any non-standard amino acids. Based on these criteria, an independent testing set of 50 human–virus protein complexes was integrated (Table S2). This docking test set was not used to develop the docking tools for the evaluation. The top level is designated as being the top *N* docking results. The accuracy, defined as the percentage of close to natural protein complex conformations among the top *N* conformations [root mean square deviation (RMSD) ≤ 10 Å], was used to assess the performance of docking tools. Other accuracies (RMSD ≤ 2 Å and RMSD ≤ 5 Å) are shown in Figure S2 and Table S2.

Nine docking tools used for protein–protein docking evaluation in the evaluation research contain three categories: seven docking tools based on FFT search algorithms (GRAMM-X, ClusPro, ZDOCK, MEGADOCK, FRODOCK, HDOCK, and CoDockPP), one docking tool based on other search algorithms (HawkDock), and one local docking tool (RosettaDock).

#### Performance of docking tools in the independent test set

**Figure 4:**
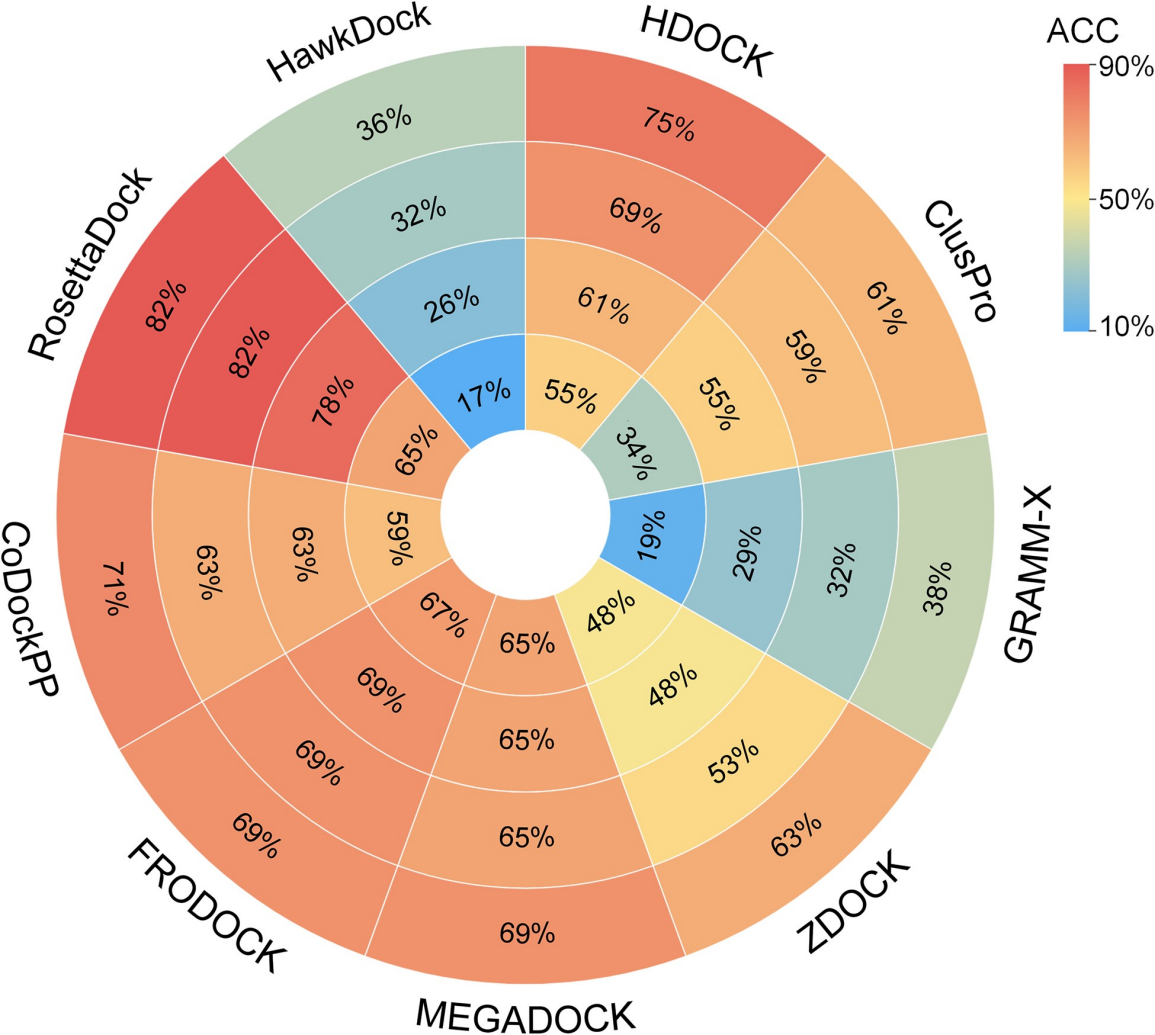
The accuracy (RMSD ≤ 10 Å) predicted at the top 1, 3, 5, and 10 levels by the docking tools The redder indicates higher accuracy and the bluer indicates lower accuracy. ACC, accuracy; RMSD, root mean square deviation.

For the overall performance of the protein–protein docking tools, as shown in **Figure 4**, FRODOCK performed the highest success rate of 67% at the top 1 level, followed by RosettaDock, MEGADOCK, CoDockPP, HDOCK, ZDOCK, ClusPro, GRAMM-X, and HawkDock. At the top 3, 5, and 10 levels, RosettaDock performed the best with success rates of 78%, 82%, and 82%, respectively, followed by FRODOCK. HawkDock did not perform well because it inherently more focuses on predicting binding free energy using MM/GBSA as well as decomposing the contribution of free energy in each residue to the binding free energy of protein complexes to help analyze the binding structure.

**Figure 5:**
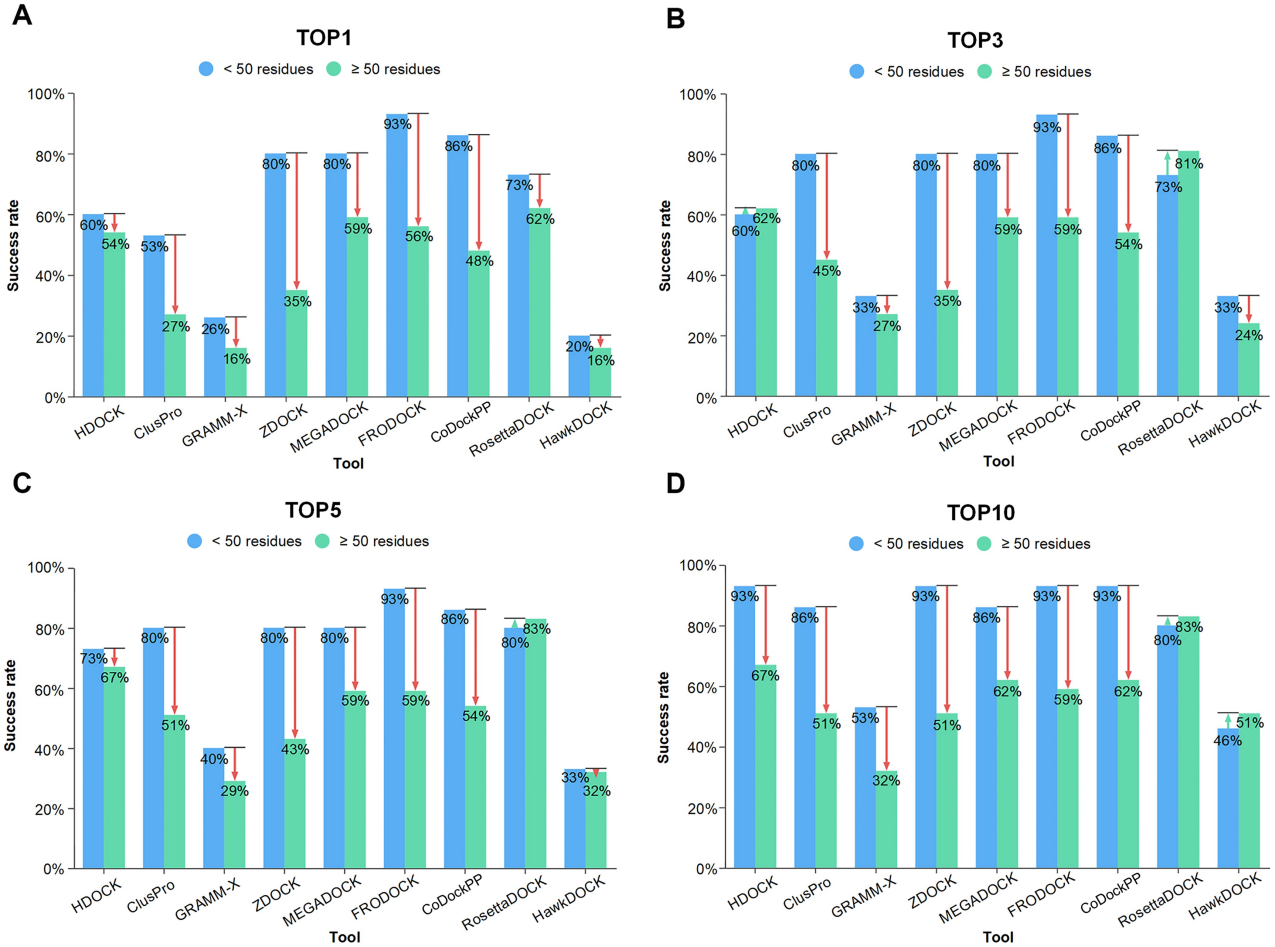
Accuracy of docking tools in the top ***N*** predictions of different viral protein length subsets **A**. Accuracy of docking tools in the top 1 predictions. **B**. Accuracy of docking tools in the top 3 predictions. **C**. Accuracy of docking tools in the top 5 predictions. **D**. Accuracy of docking tools in the top 10 predictions.

To further explore the effect of protein length on predicting the binding modes of human–virus protein complexes, the independent test set was divided into two subsets, *i.e.*, < 50 and ≥ 50 residues. As shown in **Figure 5**, we found that the docking tools as a whole performed better in the < 50 subset than in the ≥ 50 subset. FRODOCK, CoDockPP, MEGADOCK, and ZDOCK achieved outstanding predictions in the < 50 subset, with FRODOCK achieving a 93% success rate at the top 1, 3, 5, and 10 levels. However, as the viral protein becomes longer, the performance of the tested tools dropped significantly. It was worth noting that RosettaDock, HDOCK, and HawkDock had more robust performance. In particular, RosettaDock had the best prediction performance in the ≥ 50 subset, with success rates at the top 1, 3, 5, and 10 levels of 62%, 81%, 83%, and 83%, respectively.

### Opinions for improving the performance of protein–protein docking tools

Although significant progress has been made in protein–protein docking tools, there is still considerable potential for improvement as more experimentally complex structures become available. First, using FFT sampling is often followed by the need to use external scoring functions, but there is a decoupling problem between sampling and scoring, leading to a loss of accuracy. To improve performance, there is a need to optimize further or combine the available sampling and scoring strategies. Second, protein flexibility usually requires to be considered to account for interaction-induced structural rearrangements. However, flexibility can affect the accuracy of protein docking. Balancing flexibility and accuracy remains a challenging task. Third, despite significant advances in current shape complementarity functions, it simply considers the influence of neighboring atoms on grid points. Several shape-based interactions involve nearest-neighbor atoms along with numerous other non-nearest-neighbor interactions. Furthermore, with the development of algorithmic techniques and the expanding size of human–virus protein complex data, DL models are expected to be established soon for human–virus protein–protein docking.

## Conclusion and outlook

Research on human–virus PPIs contributes to a more profound knowledge of the essence of life activities and the mechanisms underlying viral infections. In addition, it provides targets for designing and developing antiviral drugs. In this review, we surveyed the available mainstream bioinformatic resources to explore human–virus PPIs, including the human–virus PPI databases, human–virus PPI computational tools, and human–virus protein docking tools, which have notable advantages in deciphering human–virus relationships. Considering the threats to human health driven by emerging viruses and the numerous unknown and suspected roles of viruses in human diseases, future studies need to reveal more virus-driven disease mechanisms by further developing bioinformatic tools to explore human–virus PPIs.

The future development of bioinformatic tools to explore human–virus PPIs is as follows. (1) More experimentally validated human–virus PPI data of distinct virus families and human–virus PPI data predicted by predictive models need to be integrated and more tightly linked to target and drug information to accelerate the translation of human–virus PPIs into novel scientific insights or applications in practice. (2) A systematic benchmarking of existing human–virus computational tools is needed. (3) AlphaFold2 [87,88], developed by the DeepMind research team, predicts most protein structures in the protein structure prediction competition CASP14 with a difference of only one atomic width from the actual structure. The tremendous progress in protein structure prediction by AlphaFold2 offers an unprecedented opportunity to develop protein bioinformatics. In addition, the emergence of AlphaFold can bring more abundant 3D structures of human–virus PPIs, which will better promote computational research on human–virus protein interactions, particularly for human–virus PPIs that are hard to predict correctly by current methods. (4) More efficient predictive tools for exploring binding patterns between human and viral proteins are needed, especially to improve performance by optimizing or combining the available sampling and scoring strategies. (5) An efficient one-stop computational pipeline is needed for data collection, prediction, and further “panoramic” analysis of the human–virus protein interactome. We hope this review will facilitate exploring human–virus PPIs based on binding modes to help decipher the human–virus relationship.

## CRediT author statement

**Huimin Chen:** Conceptualization, Formal analysis, Investigation, Resources, Writing – original draft, Writing – review & editing. **Jiaxin Liu:** Formal analysis, Investigation. **Gege Tang:** Formal analysis, Investigation. **Gefei Hao:** Conceptualization, Resources, Supervision, Writing – original draft, Writing – review & editing. **Guangfu Yang:** Conceptualization, Resources, Supervision, Writing – review & editing. All the authors have read and approved the final manuscript.

## Supplementary Material

qzae075_Supplementary_Data
