# Supplementary material for: Bioinformatic Resources for Exploring Human–virus Protein–protein Interactions Based on Binding Modes
Source: Genomics Proteomics Bioinformatics. 2024 Oct 15;22(5):qzae075. doi: 10.1093/gpbjnl/qzae075 (PMC11658832; doi:10.1093/gpbjnl/qzae075)
Supplement: qzae075_Supplementary_Data [file qzae075_supplementary_data.zip › supplementary material captions.docx]

**Supplementary** [**material**](https://www.sciencedirect.com/science/article/pii/S1672022923001031#s0195)

**Figure S1**  **The performance of the human-virus PPIs prediction tools in different virus families (each 5 data is a counting point)**

**Figure S2**  **The accuracy predicted in the top 1, 3, 5, and 10 by the docking tools**

**A.** The accuracy (RMSD ≤ 2 Å) predicted in the top 1, 3, 5, and 10 by the docking tools. **B.** The accuracy (RMSD ≤ 5 Å) predicted in the top 1, 3, 5, and 10 by the docking tools. The redder indicates higher accuracy and the bluer indicates lower accuracy.

**Table S1** **Prediction results of the human-virus PPIs prediction tools in the independent data set**

**Table S2 Summary results of the docking tools in an independent data set**
