# Supplementary material for: Bioinformatic Resources for Exploring Human–virus Protein–protein Interactions Based on Binding Modes
Source: Genomics Proteomics Bioinformatics. 2024 Oct 15;22(5):qzae075. doi: 10.1093/gpbjnl/qzae075 (PMC11658832; doi:10.1093/gpbjnl/qzae075)
Supplement: qzae075_Supplementary_Data [file qzae075_supplementary_data.zip › Table S1.docx]

**Table S1 Prediction results of the human-virus PPIs prediction tools across the entire independent data set**

| **Virus family** | **Uniprot ID** | |  | **Tool** | | | |
| --- | --- | --- | --- | --- | --- | --- | --- |
|  | **Human** | **Virus** |  | **VirusHostPPI** | **HVPPI** | **HVIDB** | **LSTM-PHV** |
| *Herpesviridae* | P33240 | P06492 |  | Yes | Yes | No | Yes |
|  | Q96RN5 | P06492 |  | Yes | No | No | Yes |
|  | P27797 | Q2HRD5 |  | Yes | Yes | Yes | Yes |
|  | Q7KZN9 | F5HGN8 |  | No | Yes | Yes | Yes |
|  | Q8IVV7 | F5HB62 |  | No | Yes | Yes | No |
|  | P05026 | F5HGN8 |  | Yes | Yes | Yes | Yes |
|  | Q96PD5 | F5HAW0 |  | No | Yes | Yes | No |
|  | P11021 | Q8AZK7 |  | Yes | Yes | Yes | Yes |
|  | Q9H7D7 | F5HB62 |  | No | Yes | Yes | Yes |
|  | Q13637 | P0CK49 |  | Yes | Yes | Yes | Yes |
|  | O43237 | F5HA27 |  | No | Yes | Yes | Yes |
|  | Q9BZG1 | G3CKS7 |  | Yes | Yes | Yes | Yes |
|  | P05067 | F5HDB7 |  | Yes | Yes | Yes | Yes |
|  | P98173 | F5HDB7 |  | No | Yes | Yes | Yes |
|  | P17612 | F5HIN0 |  | Yes | Yes | Yes | No |
|  | P20226 | F5HID2 |  | Yes | Yes | Yes | Yes |
|  | Q99615 | Q77Q38 |  | Yes | Yes | Yes | Yes |
|  | P33316 | F5H982 |  | Yes | Yes | Yes | Yes |
|  | Q9Y5M8 | P0CK58 |  | Yes | Yes | Yes | Yes |
|  | Q9H2K8 | Q2HR73 |  | No | Yes | Yes | No |
|  | Total correctly recognized PPIs | |  | 13 | 19 | 18 | 16 |
| *Papillomaviridae* | Q66K74 | P06429 |  | No | Yes | Yes | Yes |
|  | Q8WWH5 | P06464 |  | Yes | Yes | Yes | Yes |
|  | Q9NZJ0 | P03126 |  | No | No | No | No |
|  | Q9BSF8 | P03129 |  | Yes | Yes | No | No |
|  | O75528 | P03126 |  | Yes | Yes | Yes | Yes |
|  | Q13546 | P03126 |  | Yes | Yes | Yes | Yes |
|  | P68104 | P06464 |  | Yes | Yes | Yes | Yes |
|  | Q9H4P4 | P03120 |  | Yes | Yes | Yes | No |
|  | Q96DB9 | P03107 |  | No | No | No | Yes |
|  | P06733 | P06930 |  | Yes | Yes | Yes | Yes |
|  | Q9UBF8 | P06428 |  | Yes | Yes | Yes | Yes |
|  | Q13563 | B9UPF3 |  | Yes | Yes | Yes | Yes |
|  | P01106 | P06462 |  | No | No | Yes | Yes |
|  | Q9Y3C5 | P03129 |  | Yes | Yes | Yes | Yes |
|  | Q9C019 | P03129 |  | Yes | Yes | Yes | No |
|  | Q8IUD6 | P06464 |  | Yes | Yes | Yes | Yes |
|  | Q96QE5 | P24830 |  | No | Yes | Yes | Yes |
|  | O00463 | P06464 |  | Yes | Yes | Yes | Yes |
|  | Q13049 | P06464 |  | Yes | Yes | Yes | Yes |
|  | Q66K64 | P06464 |  | Yes | Yes | Yes | Yes |
|  | Total correctly recognized PPIs | |  | 15 | 17 | 17 | 16 |
| *Retroviridae* | Q99549 | P18099 |  | Yes | No | No | Yes |
|  | P37198 | P05919 |  | No | Yes | Yes | Yes |
|  | Q13885 | P18100 |  | Yes | Yes | Yes | No |
|  | Q9BVA1 | P18100 |  | Yes | Yes | Yes | No |
|  | P08779 | P18100 |  | No | Yes | No | Yes |
|  | P35527 | P18099 |  | Yes | Yes | No | Yes |
|  | P04350 | P18100 |  | Yes | Yes | Yes | Yes |
|  | Q13724 | Q77YF9 |  | Yes | No | Yes | Yes |
|  | O00571 | P18100 |  | Yes | Yes | No | No |
|  | P19388 | P05905 |  | Yes | Yes | Yes | Yes |
|  | O43660 | Q77YF8 |  | Yes | Yes | Yes | Yes |
|  | P02533 | P18100 |  | No | Yes | No | No |
|  | P35637 | P18099 |  | Yes | Yes | No | Yes |
|  | Q8N1F7 | P35967 |  | Yes | Yes | Yes | Yes |
|  | Q96P63 | P18100 |  | Yes | Yes | Yes | Yes |
|  | P13645 | P18100 |  | No | Yes | No | Yes |
|  | P31689 | P18099 |  | Yes | Yes | Yes | Yes |
|  | P06576 | P18099 |  | Yes | Yes | No | Yes |
|  | P05141 | P18099 |  | Yes | Yes | Yes | No |
|  | P68104 | P18100 |  | Yes | Yes | Yes | Yes |
|  | Total correctly recognized PPIs | |  | 16 | 18 | 12 | 15 |
| *Coronaviridae* | Q9UL15 | P59632 |  | Yes | Yes | No | Yes |
|  | Q9BU02 | A0A663DJA2 |  | No | No | No | No |
|  | Q5R372 | P0DTC3 |  | Yes | No | Yes | Yes |
|  | P04439 | P0DTC3 |  | Yes | No | No | Yes |
|  | Q9C0D3 | A0A663DJA2 |  | No | No | No | No |
|  | Q9P0L2 | P59636 |  | Yes | Yes | Yes | No |
|  | P48556 | P0DTC5 |  | Yes | No | No | Yes |
|  | Q9UL15 | P59636 |  | Yes | Yes | No | Yes |
|  | Q10713 | P0DTC5 |  | Yes | Yes | No | Yes |
|  | Q99871 | P0DTD1 |  | No | No | No | Yes |
|  | Q9BZF1 | P0DTC7 |  | Yes | Yes | No | Yes |
|  | P04439 | P0DTD8 |  | No | No | Yes | Yes |
|  | P25685 | P59634 |  | Yes | Yes | No | Yes |
|  | P40925 | P0DTC4 |  | Yes | No | No | No |
|  | Q13617 | A0A663DJA2 |  | Yes | No | No | Yes |
|  | Q3KQU3 | A0A663DJA2 |  | No | Yes | No | Yes |
|  | Q9C0C7 | Q7TLC7 |  | Yes | Yes | No | No |
|  | Q9BT22 | P0DTC7 |  | Yes | No | No | Yes |
|  | Q01813 | P0DTC7 |  | Yes | Yes | Yes | Yes |
|  | Q9BZG1 | P0DTD8 |  | Yes | No | No | Yes |
|  | Total correctly recognized PPIs | |  | 15 | 9 | 4 | 15 |
| *Flaviviridae* | Q8N1B4 | Q9WMX2 |  | Yes | Yes | Yes | Yes |
|  | Q8N5N7 | P29991 |  | No | No | Yes | Yes |
|  | Q8TBC4 | Q9WMX2 |  | Yes | Yes | Yes | No |
|  | Q9Y320 | Q99IB8 |  | Yes | Yes | No | Yes |
|  | P05141 | Q9WMX2 |  | Yes | Yes | Yes | Yes |
|  | P02675 | Q99IB8 |  | Yes | Yes | Yes | Yes |
|  | O60508 | Q32ZE1 |  | Yes | Yes | Yes | Yes |
|  | O75306 | Q9WMX2 |  | Yes | Yes | Yes | Yes |
|  | Q15629 | Q9WMX2 |  | No | Yes | Yes | No |
|  | Q9GZU8 | Q58HT7 |  | Yes | Yes | Yes | Yes |
|  | P26583 | P27958 |  | Yes | Yes | Yes | Yes |
|  | P61978 | Q6QYJ9 |  | Yes | Yes | Yes | Yes |
|  | P36542 | P29990 |  | No | Yes | Yes | Yes |
|  | Q14232 | A0A024B7W1 |  | Yes | Yes | Yes | Yes |
|  | P43246 | P29990 |  | Yes | Yes | Yes | Yes |
|  | Q00577 | P29991 |  | Yes | Yes | Yes | Yes |
|  | Q6ZT21 | Q32ZE1 |  | No | Yes | Yes | Yes |
|  | P56556 | A0A024B7W1 |  | Yes | Yes | Yes | Yes |
|  | Q15286 | A0A024B7W1 |  | Yes | Yes | Yes | Yes |
|  | P61978 | Q6QYR2 |  | Yes | Yes | Yes | Yes |
|  | Total correctly recognized PPIs | |  | 75 | 82 | 70 | 80 |
